# Validation of the 12-item World Health Organization Disability Assessment Schedule (WHODAS 2.0) in adults with attention-deficit hyperactivity disorder

**DOI:** 10.1192/bjo.2025.10873

**Published:** 2025-10-27

**Authors:** Silvia Amoretti, Juan Jesús Crespín, Montse Corrales, Carla Torrent, Derek Clougher, Santiago Biel, Carolina Ramos-Sayalero, Pol Ibáñez, Ferran Mestres, Christian Fadeuilhe, Vanesa Richarte, Josep Antoni Ramos-Quiroga

**Affiliations:** Group of Psychiatry, Mental Health and Addiction, Vall d’Hebron Research Institute (VHIR), Barcelona, Spain; Biomedical Network Research Centre on Mental Health (CIBERSAM), Barcelona, Spain; Department of Psychiatry, Vall d’Hebron University Hospital, Barcelona, Spain; Department of Psychiatry and Forensic Medicine, Autonomous University of Barcelona, Barcelona, Spain; Bipolar and Depressive Disorders Unit, Barcelona Clinical Hospital, Barcelona, Spain; Institute of Neurosciences (UBNeuro), Barcelona, Spain; Barcelona Clinical Research Foundation-August Pi i Sunyer Biomedical Research Institute (IDIBAPS), Barcelona, Spain; Department of Medicine, Faculty of Medicine and Health Sciences, University of Barcelona (UB), Barcelona, Spain; BIOARABA, Department Psychiatry, Hospital University of Alava, CIBERSAM, University of the Basque Country, Vitoria, Spain

**Keywords:** Attention deficit hyperactivity disorder, WHODAS, adult ADHD, assessment, validity

## Abstract

**Background:**

Attention deficit hyperactivity disorder (ADHD) is often associated with psychosocial functioning difficulties and valid measures of disability are needed for this population. The 12-item World Health Organization Disability Assessment Schedule 2.0 (WHODAS 2.0) is widely used to measure disability but has not been validated in the adult ADHD population.

**Aims:**

This study aims to assess the psychometric properties of the WHODAS 2.0 in adults with ADHD, and to examine differences in disability levels between ADHD subtypes and gender.

**Method:**

A cross-sectional study was conducted with 577 adults with ADHD (mean age: 38.24, s.d = 12.23; 52.3% male). ADHD severity was assessed using the ADHD Rating Scale (ADHD-RS) and Clinical Global Impression-Severity (CGI-S) Scale, while functionality was measured with the WHODAS 2.0 and the Functioning Assessment Short Test (FAST). Analyses included: (a) Cronbach’s *α* for internal consistency, (b) Pearson’s correlation for convergent validity, (c) Confirmatory Factor Analysis (CFA) for factor structure and (d) *t*-tests to compare disability levels across ADHD subtypes and gender.

**Results:**

The WHODAS 2.0 demonstrated good internal consistency (Cronbach’s *α* = 0.89). Scores were significantly correlated with psychosocial functioning (FAST, *r* = 0.476, *p* < 0.001) and clinical measures. CFA supported the original six-factor structure (root mean square error of approximation 0.039, Comparative Fit Index 0.998, Tucker–Lewis Index 0.996). When comparing ADHD subtypes, participants with the combined subtype had higher WHODAS 2.0 total scores than those with the inattentive subtype (*p* = 0.006). Additionally, gender differences were identified, with females displaying higher disability levels (*p* = 0.005).

**Conclusions:**

The WHODAS 2.0 demonstrates psychometric properties that suggest it is a valid and reliable tool for assessing disability in adults with ADHD.

Attention deficit hyperactivity disorder (ADHD) is a neurodevelopmental disorder characterised by symptoms of inattention, hyperactivity and impulsivity. ADHD symptoms typically emerge during childhood; however, around 40–50% of individuals continue to experience these ADHD symptoms in adolescence and adulthood, characterised by decreased hyperactivity but persistent inattention.^1^ Importantly, research has revealed a prevalence of around 5% in childhood^2^ and 2.5% in adults.^3^

Adults with ADHD have impaired functionality,^4^ which can negatively affect various aspects of life, including work dynamics, family life, social contacts and self-confidence.^5^ According to the literature, various factors are proposed to influence functioning, with ADHD subtypes and gender differences being particularly important.^6,7^ The functional impairments associated with ADHD are compounded by frequent comorbidities, including anxiety, depression and substance use disorders, which can further exacerbate the challenges faced by this population.^9,10^ Besides psychiatric comorbidities, individuals with ADHD also present an increased risk of physical conditions, including metabolic, cardiovascular and neurological disorders.^11^ A recent study found that individuals with the combined subtype exhibit greater functional impairments compared with those with the hyperactive/impulsive or inattentive subtype, with no significant differences between the hyperactive/impulsive and inattentive subtypes.^6^ Regarding gender differences, females with ADHD often present different symptom profiles and tend to experience more severe functional impairments, particularly in psychosocial domains.^7,8^ Individuals with ADHD show increased severity of anxiety^9^ and depression,^10^ especially females,^12^ which can further exacerbate their functional challenges.

## Measuring functional impairment

Several ADHD-specific tools have been developed to assess functional impairment in daily life. The Weiss Functional Impairment Rating Scale (WFIRS) is a widely used instrument that evaluates functioning across domains particularly relevant to ADHD, including family, school/work, life skills, self-concept, social activities and risky behaviour.^13^ Similarly, the Sheehan Disability Scale (SDS) is frequently used as a brief self-report tool designed to assess functional impairment across key life areas – such as work or school, social activities and family responsibilities – affected by psychiatric symptoms.^14^ These instruments focus on ADHD-specific domains of impairment and are valuable in characterising the disorder’s functional impact. However, they may be limited in capturing broader aspects of disability that extend beyond ADHD-specific challenges. To address this gap, the World Health Organization (WHO) emphasises that disability and functioning should be assessed using measures conceptually and operationally linked to the framework of the International Classification of Functioning, Disability and Health (ICF).^15^ In line with this framework, the 12-item World Health Organization Disability Assessment Schedule 2.0 (WHODAS 2.0) was developed and is a commonly used tool for measuring disability across various health conditions. WHODAS 2.0 has been utilised as a standard measure in global health research, providing a benchmark for comparing functional impairments across different populations and health conditions.^16^ The adaptation of this assessment is derived from the 36-item version to provide a shorter tool for assessing overall functioning through surveys or health outcome studies. Its adequate reliability has been highlighted, explaining 81% of the variability observed in the full version of the WHODAS 2.0.^16^ The WHODAS 2.0 addresses many aspects, including a broader perspective that encompasses cognition, self-care, mobility, interpersonal relationships and daily life activities, both personal and professional. Despite the fact that it can be used to assess disability in adults suffering from any illness, whether somatic, mental or substance-related,^16^ it has not been validated in the adult ADHD population, indicating a significant need for validation. Validating the WHODAS 2.0 in adults with ADHD is vital for ensuring that the tool accurately reflects the specific challenges faced by this population, thereby enhancing its utility in both clinical and research settings.

### Aims

Therefore, the primary aim of this study is to examine the psychometric properties of the WHODAS 2.0 questionnaire in a sample of adults with ADHD. Additionally, the study examines differences in WHODAS 2.0 scores across specific subgroups, such as ADHD subtypes and gender, to assess the distinct functional impairments associated with each, thereby contributing to more personalised interventions in clinical practice.

## Method

### Design of the study and patients

This observational cross-sectional study was carried out within the adult ADHD Programme of the Psychiatry Department of the Vall d’Hebron University Hospital in Barcelona (Spain). Participant recruitment was conducted consecutively between 2019 and 2024. The study followed the ethical standards outlined in the Declaration of Helsinki of 1975, as revised in 2013, and complied with Good Clinical Practice guidelines. Ethical approval was obtained from the Clinical Research Ethics Committee of Vall d’Hebron University Hospital (PR(AG)103/2019). All participants provided written informed consent and were not compensated financially for their participation.

The following inclusion criteria were established: ≥18 years of age, provide informed consent and meet the diagnostic criteria for ADHD according to the DSM-5.

### Instruments and procedures

The clinical diagnosis of ADHD was made by senior psychiatrists and psychologists according to the criteria established by the DSM-5. The ADHD diagnosis was evaluated and confirmed using the Conners’ Adult ADHD Diagnostic Interview for DSM-IV (CAADID)^17^ and the Diagnostic Interview for ADHD in Adults (DIVA 2.0).^18^ The CAADID, a semi-structured interview, assesses ADHD symptoms, while the DIVA 2.0 evaluates the diagnostic criteria.

The ADHD Rating Scale (ADHD-RS)^19^ and the Clinical Global Impression Severity Scale (CGI-S)^20^ were used to evaluate the clinical severity of ADHD. The Wender Utah Rating Scale (WURS)^21^ was used to assess a range of childhood symptoms and behaviours indicative of ADHD that persist into adulthood. Further, to ensure a systematic and standardised evaluation of psychiatric comorbidity, participants were assessed using the Structured Clinical Interview for DSM-IV (SCID-I and II).^22,23^ Depressive symptoms were measured with the Beck Depression Inventory II (BDI-II),^24^ while anxiety levels were assessed using the State-Trait Anxiety Inventory (STAI),^25^ which captures both trait and state components. Impulsivity was evaluated with the Barratt Impulsiveness Scale (BIS-11)^26^ which provides a total score as well as scores across three distinct dimensions: cognitive impulsivity, motor impulsivity and non-planning impulsivity. Participants completed self-report measures at home, ensuring they had sufficient time to reflect on their responses. Any questions or uncertainties about the measures were addressed during the subsequent evaluation session, ensuring clarity and completeness of data collection.

Functional impairment was evaluated using two instruments: the Functioning Assessment Short Test (FAST)^4,27^ and the 12-item version of the World Health Organization Disability Assessment Schedule 2.0 (WHODAS 2.0).^16^ The FAST consists of 24 items, each item rated from 0 (no difficulty) to 3 (severe difficulty), covering 6 key domains of daily functioning: autonomy, occupational functioning, cognitive functioning, financial issues, interpersonal relationships and leisure time. Higher total scores reflect greater impairment. WHODAS 2.0 assesses disability across six domains aligned with the International Classification of Functioning, Disability and Health (ICF) framework: cognition (e.g. understanding and communication), mobility (e.g. moving and walking), self-care (e.g. hygiene, dressing and eating), interpersonal relationships, life activities (including domestic and work-related tasks) and participation in society. Each domain is evaluated using two items and higher scores indicate greater disability.

To maintain consistency across evaluations, all assessments were conducted by the same team of trained clinicians throughout the study period. This ensured standardisation in the administration and scoring of all measures.

### Statistical analysis

The required sample size was estimated based on the psychometric study by Abdin et al (2023),^28^ ensuring statistical power of 80% and a significance threshold of *α* = 0.05. According to this calculation, a minimum of 233 participants was necessary.

Descriptive statistics were computed for all study variables, including mean, s.d. and percentage as appropriate. To evaluate the internal consistency of the WHODAS 2.0, Cronbach’s *α* coefficients were calculated. Concurrent validity was examined through Pearson’s correlations between WHODAS 2.0 scores and clinical measures. To assess the underlying factorial structure of the WHODAS 2.0, Confirmatory Factor Analysis (CFA) was conducted, evaluating model fit using standard indices: a Comparative Fit Index (CFI) and Tucker–Lewis Index (TLI) above 0.90, and a root mean square error of approximation (RMSEA) below 0.10 were considered indicative of good fit.^29–32^ Differences in WHODAS 2.0 scores between ADHD subtypes and between genders were analysed using independent sample *t*-tests. Effect sizes were calculated to estimate the magnitude of observed differences.

All statistical analyses were performed using IBM SPSS Statistics version 26 for Windows (IBM Corp., Armonk, NY, USA; see https://www.ibm.com/analytics/spss-statistics-software). Significance was set at a two-tailed *p*-value of <0.05.

## Results

### Demographic and clinical characteristics of the sample

A total of 577 subjects with ADHD were recruited for the study, with a mean age of 38.24 years (s.d. = 12.23). The sample had a nearly balanced gender distribution, with males representing 52.3% of the participants. The average age at which ADHD was diagnosed was 26.55 years (s.d. = 15.41).

Regarding employment status, 52.9% of the participants were actively employed, 12.3% were actively seeking employment and 22.2% were students. The remaining participants were either disabled (2.1%) or on sick leave (3.5%). In terms of substance use, 34.3% of the participants consumed alcohol, 28.2% smoked tobacco and 18.9% used cannabis.

### Internal consistency

The internal consistency of WHODAS was assessed using Cronbach’s *α* coefficient, which was found to be 0.89 (90% CI: 0.87–0.90), indicating a good internal consistency.

### Concurrent validity

**Fig. 1:**
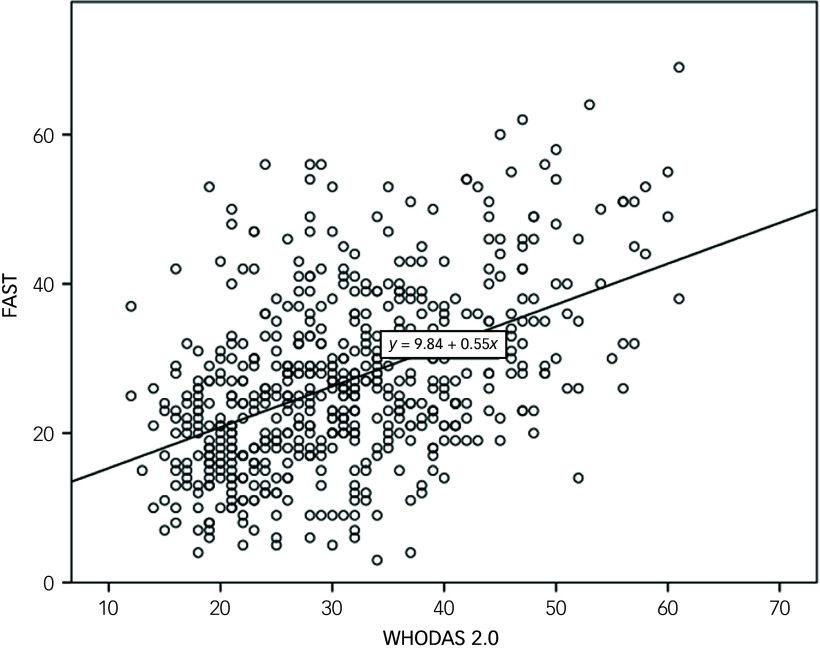
Scatter plot and regression line of correlations between the total sum of the 12-item version of World Health Organization Disability Assessment Schedule (WHODAS) 2.0 and the Functioning Assessment Short Test (FAST).

The concurrent validity of the WHODAS 2.0 scale was assessed through correlations with various clinical outcomes. WHODAS 2.0 scores showed positive correlations with psychosocial functioning (FAST scale, *r* = 0.476, *p* < 0.001) (Fig. 1) and several clinical scales, including the ADHD-RS (*r* = 0.394, *p* < 0.001), CGI-S (*r* = 0.306, *p* < 0.001), BDI-II (*r* = 0.611, *p* < 0.001), STAI (state, *r* = 0.523, *p* < 0.001; trait, *r* = 0.567, *p* < 0.001) and BIS (*r* = 0.343, *p* < 0.001).

### Construct validity

**Fig. 2:**
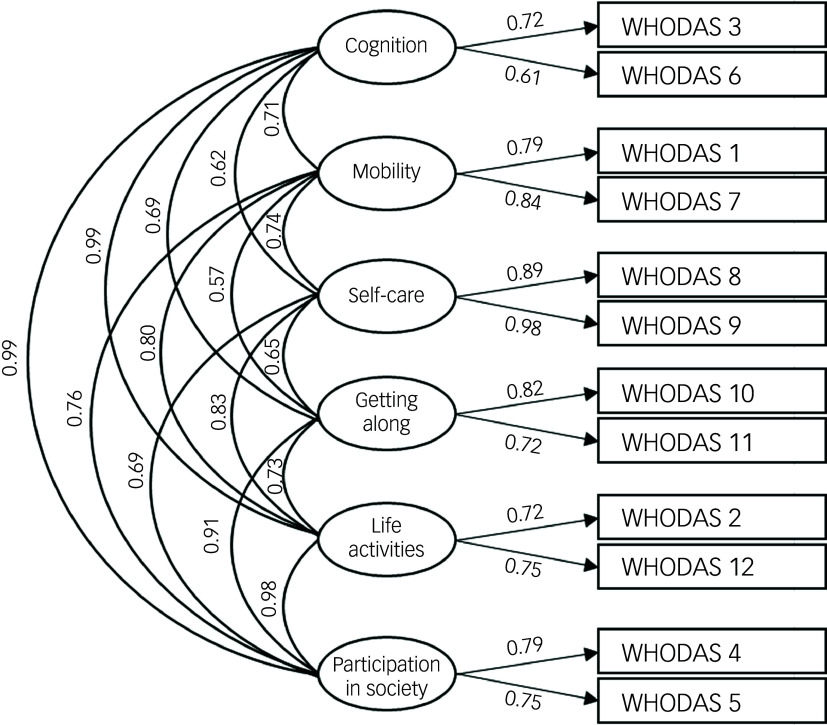
Confirmatory factor analysis, using 12 items of the original WHODAS 2.0 scale. WHODAS 1: Standing for long periods such as 30 min; WHODAS 2: Taking care of your household responsibilities; WHODAS 3: Learning a new task, such as learning how to get to a new place; WHODAS 4: How much of a problem do you have joining in community activities (for example, festivities) in the same way as anyone else can; WHODAS 5: How much have you been emotionally affected by your health problems; WHODAS 6: Concentrating on doing something for ten minutes; WHODAS 7: Walking a long distance such as a kilometre or equivalent; WHODAS 8: Washing your whole body; WHODAS 9: Getting dressed; WHODAS 10: Dealing with people you do not know; WHODAS 11: Maintaining a friendship; WHODAS 12: Your day-to-day work/school.

To assess the construct validity of the WHODAS 2.0, a CFA was conducted using the diagonally weighted least squares (DWLS) estimation method. All item loadings were statistically significant (*p* < 0.05) and ranged from 0.61 to 0.99, indicating strong contributions of the individual items to their respective factors. The six-factor model, corresponding to the original WHODAS 2.0 structure, demonstrated a good fit with the data (Fig. 2). Model fit indices supported the adequacy of the proposed structure, with a RMSEA value of 0.039 (90% CI [0.024, 0.051]), a CFI value of 0.998 and a TLI value of 0.996.

### ADHD types

The analysis of ADHD types revealed significant differences between the combined and inattentive subtypes in several domains. The WHODAS 2.0 total score was significantly higher in the combined subtype (*p* = 0.006), with differences in domains such as mobility (*p* = 0.018) and participation in society (*p* = 0.006) (see Table 1).

**Table 1:** Comparison of sociodemographic features, clinical characteristics and the World Health Organization Disability Assessment Schedule (WHODAS) 2.0 scale between combined and inattentive attention-deficit hyperactivity disorder (ADHD) subtypes

	Combined (*n* = 299)	Inattentive (*n* = 278)	*t* or *χ*²	*p*	Effect size
Sociodemographic features					
Age, years	39.48 ± 12.20	36.92 ± 12.15	2.525	**0.012**	0.210
Gender (females)	145 (48.7)	126 (45.8)	0.463	0.504	0.028
Occupation (working)	161 (57.9)	144 (55.8)	20.593	**<0.001**	0.196
Alcohol (yes)	126 (42.6)	72 (26.2)	16.897	**<0.001**	0.172
Tobacco (yes)	105 (35.5)	58 (21.1)	14.456	**<0.001**	0.159
Cannabis (yes)	77 (26)	32 (11.6)	19.078	**<0.001**	0.183
Clinical characteristics					
Age at onset of ADHD symptoms, years	15.21 ± 13.79	14.00 ± 11.28	0.529	0.597	0.096
Age of ADHD diagnosis, years	26.50 ± 15.68	26.60 ± 15.23	−0.055	0.956	0.006
WURS	56.05 ± 21.90	48.52 ± 17.81	4.437	**<0.001**	0.377
ADHD-RS	32.10 ± 8.33	28.57 ± 8.17	5.107	**<0.001**	0.428
CGI	4.15 ± 0.81	3.77 ± 0.76	4.919	**<0.001**	0.484
Depressive symptoms (BDI)	18.88 ± 11.71	16.05 ± 10.60	3.028	**0.003**	0.253
Anxiety trait (STAI)	31.22 ± 13.66	28.65 ± 12.59	2.327	**0.020**	0.196
Anxiety state (STAI)	35.24 ± 12.00	33.03 ± 11.05	2.274	**0.023**	0.192
Impulsivity (BIS-11)	72.50 ± 14.56	64.33 ± 15.45	6.472	**<0.001**	0.544
FAST	27.45 ± 11.95	26.13 ± 11.36	1.347	0.178	0.113
WHODAS					
WHODAS total score	32.13 ± 10.39	29.81 ± 9.75	2.761	**0.006**	0.230
Cognition	3.19 ± 1.85	2.90 ± 1.87	1.776	0.076	0.156
Mobility	1.98 ± 2.05	1.58 ± 2.01	2.371	**0.018**	0.197
Self-care	0.99 ± 1.64	0.85 ± 1.56	1.053	0.293	0.087
Getting along	2.40 ± 2.14	2.17 ± 2.12	1.295	0.196	0.108
Life activities	3.44 ± 2.11	3.35 ± 2.07	0.517	0.605	0.043
Participation in society	3.28 ± 2.32	2.75 ± 2.18	2.771	**0.006**	0.235

Data are presented as *n* (%) or mean ± s.d.

WURS, Wender Utah Rating Scale; ADHD-RS, ADHD Rating Scale; CGI, Clinical Global Impression Scale; BDI, Beck-II Depression Inventory II; STAI, State-Trait Anxiety Inventory; BIS-11, Barratt Impulsiveness Scale; FAST, Functioning Assessment Short Test; WHODAS, World Health Organization Disability Assessment Schedule 2.0.

Significant differences (*p* < 0.05) marked in bold.

Participants with the combined type of ADHD had a higher prevalence of substance use, with significant differences observed in alcohol (*p* < 0.001), tobacco (*p* < 0.001) and cannabis use (*p* < 0.001). Additionally, those with the combined type showed higher scores on the WURS (*p* < 0.001), ADHD-RS (*p* < 0.001) and CGI (*p* < 0.001). Psychometric measures also indicated higher levels of depressive symptoms (BDI, *p* = 0.003), anxiety (STAI-trait, *p* = 0.020; STAI-state, *p* = 0.023) and impulsivity (BIS-11, *p* < 0.001) in the combined group.

### Gender differences

Both the WHODAS 2.0 total score (*p* = 0.005) and all subdomains, except for getting along (*p* = 0.084), were significantly higher in females than in males.

Females had a higher mean age (*p* < 0.001) and age of ADHD diagnosis (*p* < 0.001) compared with males, with no differences in terms of the age at onset for ADHD symptoms (*p* = 0.937). Females reported higher levels of depressive symptoms (BDI, *p* = 0.002), anxiety (STAI-trait, *p* = 0.003; STAI-state, *p* < 0.001) and functional impairment (FAST, *p* = 0.049). All details can be found in Table 2.

**Table 2:** Clinical and sociodemographic characteristics of attention-deficit hyperactivity disorder (ADHD) patients included in the study according to gender

	Females (*n* = 273)	Males (*n* = 304)	*t* or *χ*²	*p*	Effect size
Sociodemographic features					
Age, years	40.34 ± 12.24	36.40 ± 11.93	3.902	**<0.001**	0.326
Occupation (working)	167 (61.3)	161 (53.1)	4.227	0.376	0.001
Alcohol (yes)	83 (30.3)	118 (38.7)	4.444	**0.022**	0.088
Tobacco (yes)	73 (26.6)	92 (30.3)	0.990	0.184	0.042
Cannabis (yes)	44 (16.2)	66 (21.7)	2.719	0.061	0.069
Clinical characteristics					
ADHD (combined type)	146 (53.5)	154 (50.7)	0.463	0.275	0.028
Age at onset of ADHD symptoms, years	14.67 ± 12.63	14.85 ± 13.22	−0.079	0.937	0.014
Age of ADHD diagnosis	30.04 ± 15.17	23.98 ± 15.12	3.558	**<0.001**	0.400
WURS	53.38 ± 20.53	51.65 ± 20.18	0.997	0.319	0.085
ADHD-RS	31.65 ± 8.82	29.41 ± 7.90	3.182	**0.002**	0.268
CGI	4.01 ± 0.81	3.93 ± 0.80	0.126	0.088	0.099
Depressive symptoms (BDI)	19.08 ± 11.29	16.11 ± 11.14	3.149	**0.002**	0.265
Anxiety trait (STAI)	31.74 ± 13.37	28.44 ± 12.94	2.974	**0.003**	0.251
Anxiety state (STAI)	36.29 ± 11.46	32.32 ± 11.43	4.104	**<0.001**	0.347
Impulsivity (BIS-11)	69.24 ± 14.82	67.98 ± 16.20	0.953	0.341	0.081
FAST	27.85 ± 11.41	25.90 ± 11.91	1.976	**0.049**	0.167
WHODAS					
WHODAS total score	32.29 ± 10.58	29.90 ± 9.65	2.806	**0.005**	0.236
Cognition	3.23 ± 1.93	2.90 ± 1.80	2.116	**0.035**	0.177
Mobility	2.16 ± 2.19	1.46 ± 1.83	4.066	**<0.001**	0.347
Self-care	1.25 ± 1.91	0.63 ± 1.20	4.563	**<0.001**	0.389
Getting along	2.45 ± 2.17	2.14 ± 2.09	1.730	0.084	0.146
Life activities	3.73 ± 2.20	3.08 ± 1.93	3.765	**<0.001**	0.314
Participation in society	3.32 ± 2.30	2.76 ± 2.21	2.938	**0.003**	0.248

Data are presented as *n* (%) or mean ± s.d.

WURS, Wender Utah Rating Scale; ADHD-RS, ADHD Rating Scale; CGI, Clinical Global Impression Scale; BDI, Beck-II Depression Inventory II; STAI, State-Trait Anxiety Inventory; BIS-11, Barratt Impulsiveness Scale; FAST, Functioning Assessment Short Test; WHODAS, World Health Organization Disability Assessment Schedule 2.0.

Significant differences (*p* < 0.05) marked in bold.

## Discussion

The main finding of this study is that the WHODAS 2.0 demonstrates strong psychometric properties suggesting it is a valid and reliable tool for assessing disability in adults with ADHD. While previous research has underscored the reliability and utility of WHODAS 2.0 across various mental health conditions.^28,33,34^ This study is, to our knowledge, the first to focus specifically on the validation of WHODAS 2.0 in adults with ADHD, thereby addressing an important gap in the literature regarding the use of transdiagnostic disability measures in this population.

Cronbach’s *α* coefficient of 0.89 demonstrates that WHODAS 2.0 offers a reliable measure of functional impairment. This level of internal consistency aligns with previous research conducted in various clinical populations, such as anxiety and stress disorders (Cronbach’s *α* ranging from 0.83 to 0.92),^35^ autism spectrum disorder (Cronbach’s *α* = 0.86),^36^ Huntington’s disease (Cronbach’s *α* = 0.94),^31^ first major depressive episode (Cronbach’s *α* = 0.89),^37^ psychotic disorder (Cronbach’s *α* = 0.89)^34^ and other conditions including schizophrenia, depression, anxiety, and diabetes (Cronbach’s *α* = 0.88–0.91)^28^ and chronic diseases.^38^ Furthermore, concurrent validity was supported by a significant correlation between a WHODAS 2.0 score and a FAST scale, which assesses functionality. Moreover, as expected, positive correlations were also found with other clinical measures. Higher severity (measured by the ADHD-RS and CGI-S), as well as increased depressive (BDI-II), impulsive (BIS-11) and anxious (STAI) symptoms, were significantly associated with greater levels of disability.

CFA provided strong evidence for the six-factor structure of WHODAS 2.0,^16^ which included six core domains: (a) cognition (understanding and communication); (b) mobility (the ability to move and navigate); (c) self-care (managing personal hygiene, dressing, eating and independent living); (d) getting along (interpersonal interactions); (e) life activities (responsibilities in home, work or school environments) and (f) participation in society (involvement in community and recreational activities). The validation of the 6-factor structure in the 12-item version is consistent with previous studies, such as Carlozzi et al (2015), and also mirrors the validation of the 36-item version.^39–41^ This consistency across versions and clinical populations underscores the robustness of WHODAS 2.0 as a measure of disability.

Significant differences in global disability scores, particularly in mobility and participation in society, were observed between the combined and inattentive ADHD subtypes, with individuals with the combined subtype exhibiting greater impairments. They also presented higher clinical severity. These findings are consistent with prior research indicating that individuals with the combined subtype experience more severe impairments compared to those with the inattentive or hyperactive/impulsive subtypes.^6^ Individuals with the combined type were also more likely to engage in substance use and experience higher levels of comorbid symptoms such as anxiety and depression.^42^ Thus, it seems that these comorbid factors likely contribute to the overall disability burden.

Significant gender differences in WHODAS 2.0 scores were observed, indicating distinct functional challenges faced by males and females with ADHD. Females reported higher levels of functional impairment across nearly all WHODAS 2.0 domains. This finding is consistent with earlier studies^7^ suggesting that females with ADHD often exhibit more severe impairments and experience higher rates of comorbid anxiety and depression.^43^ While both females and males reported a similar age at onset for ADHD symptoms, there is a notable difference in the timing of diagnosis: females were diagnosed with ADHD significantly later than males (30.04 ± 15.17 *v*. 23.98 ± 15.12). This diagnostic delay may be related to differences in symptom presentation or under-recognition of ADHD in women, potentially due to societal expectations, gender biases or symptomatic differences that mask ADHD symptoms, as discussed in recent literature.^44^ The later diagnosis in females could contribute to increased depressive symptoms, anxiety and functional impairment as suggested by recent studies.^44^ These findings suggest that the later diagnosis in females may lead to prolonged struggles with untreated ADHD, potentially exacerbating comorbid conditions and functional impairments. Therefore, addressing these diagnostic and symptomatic differences is crucial for improving outcomes in females with ADHD.

Beyond its psychometric strengths, the validation of WHODAS 2.0 in adults with ADHD has relevant clinical and research implications. Clinically, it provides a brief, standardised and transdiagnostic tool to assess disability across multiple domains of functioning, supporting the development of individualised treatment plans and the monitoring of patient progress over time. From a research perspective, WHODAS 2.0 facilitates comparisons across diagnostic groups and health conditions, making it particularly valuable in transdiagnostic, longitudinal or epidemiological studies. Its alignment with the ICF framework also promotes consistency in outcome measurement and enhances its potential for integration into global mental health initiatives.

Several limitations of this study should be considered before translating the findings into clinical practice. First, the cross-sectional design limits the ability to assess changes in disability over time, which would be valuable for understanding how interventions might influence long-term outcomes. Second, the absence of a control group restricts the ability to compare disability levels between individuals with ADHD and the general population. Third, the recruitment of participants from a single clinical site may limit the generalisability of the findings to broader ADHD populations. Fourth, although concurrent validity was assessed using the FAST scale, the study did not include other instruments that have been used to assess functional impairment in adults with ADHD. As a result, the criterion validity of WHODAS 2.0 could not be explored in relation to ADHD-specific measures, which should be considered in future research. Finally, future studies could address these issues by employing a longitudinal design and including a more diverse participant pool, allowing for a more nuanced understanding of functional impairment across different contexts and life stages. Despite these limitations, the study benefits from a large sample size, which enabled robust analyses of ADHD subtypes and gender differences in disability. Additionally, the inclusion of multiple validated instruments, such as the ADHD-RS, BDI-II and FAST, strengthened the analysis and ensured a comprehensive evaluation of both symptom severity and functional outcomes.

In conclusion, the findings of this study suggest that the WHODAS 2.0 demonstrates psychometric properties indicating it is a valid and reliable tool for assessing disability in adults with ADHD. These results provide valuable insights into the differences across ADHD subtypes and between genders. The study underscores the importance of improving early diagnoses and developing tailored interventions that address the specific needs of individuals based on their ADHD presentation and gender, thereby providing a foundation for more personalised care strategies.

## Data Availability

The data that support the findings of this study are available on request from the corresponding authors.

## Data confidentiality

All the protocols of our centre were followed to maintain the confidentiality of the data. No patient data appear in this article.
